# Development of fatty acid analogues with potent anabolic effects on bone in male mice

**DOI:** 10.1016/j.bonr.2025.101862

**Published:** 2025-07-30

**Authors:** Jian-ming Lin, Ivo Dimitrov, Karen E. Callon, Maureen Watson, Ian R. Reid, William A. Denny, Jillian Cornish

**Affiliations:** a Department of Medicine, The University of Auckland, Auckland, New Zealand; b Auckland Cancer Society Research Centre, School of Medical Sciences, The University of Auckland, Auckland, New Zealand

**Keywords:** Fatty acid, Analogues, Osteoclast, Osteoblast, Bone resorption, Bone formation

## Abstract

Natural fatty acids are inhibitory to osteoclastogenesis, but only mildly so, as reported earlier by our and other groups. To improve the potency, we have synthesized two categories of analogues based on the backbone of saturated palmitic acid by inserting an ether or a triazole group in the carbon chain. The most effective compound proved to be with a triazole moiety farthest away from the acid unit. Following this strategy, we now have developed even more potent molecules, methylated triazole and tetrazole analogues. Tetrazole analogue displays about 10-fold higher inhibitory activity over the natural counterpart as tested in the osteoclastogenesis assay using mouse bone marrow cell cultures. Importantly, this inhibition is not due to cytotoxicity as both the methylated triazole and tetrazole molecules slightly increase the viability of bone marrow cells. It was found that the inhibition of osteoclastogenesis by the tetrazole analogue in mouse bone marrow cultures is associated with the decreased expression of the key osteoclastogenic or osteoclastic marker genes: *Dcstamp*, *Nfatc1*, *Tnfa*, *Trap* and *Ctsk*. The best analogue-tetrazole was then tested *in vivo* in a mouse calvarial local injection model after being solubilized by (2-hydroxypropyl)-β-cyclodextrin (β-CD). The results show that the tetrazole at the daily dose of 40 μg/injection (along with 264 μg β-CD) significantly reduce TRAP surface, and significantly increased mineralizing surface/bone surface, mineral apposition rate and bone formation rate. This study provides a novel effective agent for inhibiting osteoclastogenesis and positively regulating bone homeostasis.

## 1 Introduction

Bone is a dynamic organ undergoing constant remodelling. The balance of bone homeostasis is dependent on the activity of the bone-forming osteoblasts and bone-resorbing osteoclasts. Excess of bone resorption over bone formation leads to lower bone mass or osteoporosis, which is a common disorder affecting 200 million people worldwide (Reginster and Burlet, 2006). Therefore, inhibition of osteoclast formation or/and function is one of the strategies to positively regulate bone balance.

Fatty acids, in the form of triglycerides, are a major dietary component, comprising ~4% of dairy milk (Jensen, 2002) and 20–35% of calories in the daily diet intake in humans (Liu et al., 2017). Their effect on bone has been a focus of study in the field. We have previously found that fatty acids are inhibitory to osteoclastogenesis and bone resorption activity *in vitro* (Cornish et al., 2008). This observation has not only provided a novel linkage for the positive correlation between fat mass and bone mass, but also has revealed a new category of nutrient components with therapeutic potential in treating osteoporosis. However, some drawbacks of fatty acids have complicated their development for clinical applications. Firstly, their activity in osteoclast inhibition is modest, likely due to their rapid esterification and oxidation (Luiken et al., 1997; Rioux et al., 2000). Secondly, they are not aqueously soluble and have to be dissolved in organic solvents, which are toxic to cells and animals.

To overcome these issues, we have previously synthesized two categories of analogues by inserting either an ether or a triazole group into the backbone of the 16-carbon chain. Among the analogues synthesized, the one with the triazole group farthest away from the carboxylic acid group displayed highest osteoclast inhibition activity, with a several-fold higher activity than the natural palmitic acid in the assays *in vitro* (Marshall et al., 2013). This progress has encouraged us to seek an analogue with even higher potency. At this stage, significant improvement has been achieved with the production of two new compounds, methylated triazole and tetrazole analogues, the latter of which displays a greater than ten-fold activity in comparison to the natural fatty acid in inhibiting osteoclasts in mouse bone marrow culture. However, this analogue is still not adequately water-soluble. Thus, the analogue was complexed with a carrier (2-hydroxypropyl)-β-cyclodextrin (β-CD) producing an inclusion complex that could be dissolved in water enabling the use of it in a calvarial local injection model to test its effect on bone *in vivo*. β-CD contains a hydrophobic cavity to accommodate the compound and a hydrophilic surface to disperse in water. It is non-toxic and does not result in an immune response in humans, and is widely used in food, cosmetic and pharmaceutical products (Kurkov and Loftsson, 2013; Davis and Brewster, 2004; Fenyvesi et al., 2016). In this *in vivo* study, the tetrazole analogue displayed anabolic effects, suggesting the potential to develop it into a pharmaceutical agent for treating osteoporosis.

## 2 Materials and methods

### 2.1 Animals and cell culture

αMEM, DMEM, MEM and fetal bovine serum (FBS) were purchased from Thermo Fisher, NZ. The cells were cultured in an incubator with a humidified atmosphere and 5% CO_2_ at 37°C. All culture media contain 100 U/mL of penicillin and 100 μg/mL streptomycin, which were purchased from Life Technologies. The studies involving the use of animals were approved by the Animal Ethical Committee of the University of Auckland.

### 2.2 Analogue synthesis

**Fig. 1:**
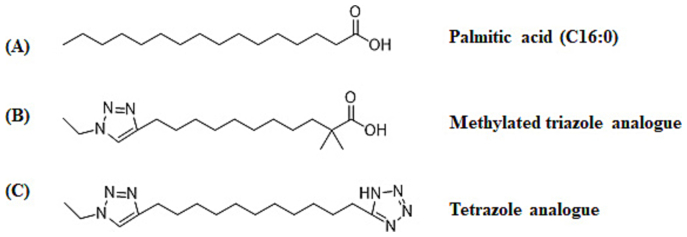
Chemical structure of palmitic acid and its analogues. A: palmitic acid, the backbone for the analogues synthesized; B: methylated triazole analogue; C: tetrazole analogue.

The structures of the analogues methylated triazole and tetrazole are shown in Fig. 1. The procedure for the synthesis of the analogues is as follows.

**Figure:**
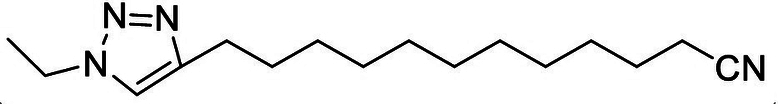

12-(1-ethyl-1*H*-1,2,3-triazol-4-yl)dodecanenitrile

Triethyl amine (0.11 g, 1.04 mmol) was added to a solution of 11-(1-ethyl-1*H*-1,2,3-triazol-4-yl)undecan-1-ol (0.14 g, 0.52 mmol) in DCM (5 mL) at 0°C (Marshall et al., 2013). Methanesulfonyl chloride (0.09 g, 0.79 mmol) was added dropwise to the solution. The reaction was stirred for 2 h at 0°C. The reaction mixture was washed with ice cold water and concentrated *in vacuo.* The resulting crude oil was taken up in DMSO (5 mL) and KCN (0.1 g, 1.60 mmol) was added.

The resulting solution was stirred overnight at room temperature. The reaction mixture was then quenched with water (5 mL), extracted with EtOAc (3 × 10 mL). The organic layers were dried over MgSO_4_ and concentrated. Purification with flash chromatography eluting with DCM/EtOAc (10–20%) yielded the title compound (0.06 g, 42%) as a yellow oil.

^1^HNMR (DMSO) δ 7.83 (s, 1H), 4.34–4.27(q, *J* = 7.32 Hz, 2H), 2.66–2.62 (t, *J* = 7.80 Hz, 2H), 2.49–2.45 (t, *J* = 7.0 Hz, 2H), 1.58–1.51 (m, 4H), 1.41–1.38 (t, *J* = 7.32 Hz, 3H), 1.27–1.20 (m, 14H); MS *m/z* 277.30 (MH)^+^.

**Figure:**
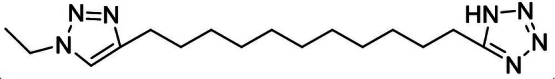

5-(11-(1-ethyl-1*H*-1,2,3-triazol-4-yl)undecyl)-1H-tetrazole

NaN_3_ (0.028 g, 0.44 mmol) and Et_3_N.HCl (0.06 g, 0.44 mmol) were added to a solution of 12-(1-ethyl-1*H*-1,2,3-triazol-4-yl)dodecanenitrile (0.04 g, 0.15 mmol) in DMF (5 mL), the reaction mixture was heated in a microwave to a 130°C over 24 h. The reaction mixture was cooled to room temperature, quenched with water (5 mL) and extracted with EtOAc (3 × 10 mL). The organic layers were dried over MgSO_4_ and concentrated. Purification with flash chromatography eluting with DCM/EtOAc (50%) yielded the title compound (0.023 g, 50%) as a yellow oil.

^1^HNMR (CDCl_3_) δ 7.53 (s, 1H), 4.44–4.38 (q, *J* = 7.32 Hz. 2H), 3.05–2.99 (t, *J* = 7.6 Hz, 2H), 2.72–2.68 (t, *J* = 7.08 Hz, 2H), 1.59–1.53 (m, 11H), 1.53–1.50 (t, *J* = 7.32 Hz, 3H), 1.27–1.24 (m, 7H); ^13^CNMR (CDCl_3_) δ 119.98, 45.26, 33.01, 29.92, 29.73, 29.65, 29.59, 29.54, 29.64, 28.94. 28.86, 25.93, 25.57, 17.34, 15.77; MS *m/z* 319.30 (MH)^+^, Calculated for C_16_H_29_N_7_ (MH^+^) 320.25572, found 320.25724.

**Figure:**
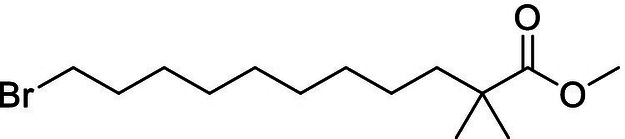

methyl 11-bromo-2,2-dimethylundecanoate

To a solution of diisopropylamine (4.9 g, 49 mmol) in THF (25 mL) cooled to 0°C was added *n*BuLi (24.5 mL, 49 mmol) and the mixture was stirred at 0°C for 30 min. The reaction mixture was then cooled to −78°C, methyl isobutyrate (5 g, 49 mmol) was added dropwise and the stirring was continued for 1 h at −78°C. 1,9-Dibromononane (14.2 g, 49.5 mmol) was added dropwise and the reaction continued to be stirred at −78°C for an additional hour. The reaction mixture was then warmed to room temperature over 2 h. The reaction was then quenched with saturated aqueous ammonium chloride and extracted with EtOAc (2 × 25 mL). The extracts were then washed with 1 M HCl (aq.) (2 × 20 mL) and brine. The organic extracts were dried over MgSO_4_ and concentrated. Purification with flash chromatography eluting with Hexanes/EtOAc(0–3%) yielded the title compound (10.8 g, 72%) as a yellow oil.

^1^HNMR (CDCl_3_) δ 3.66 (s, 3H), 3.42–3.39 (t, *J* = 6.8 Hz, 2H), 1.88–1.81 (q, *J* = 6.93 Hz, 2H), 1.51–1.47 (m, 2H), 1.43–1.41 (m, 2H), 1.27–1.18 (m, 10H), 1.15 (s, 6H); MS *m/z* 307.20 (MH)^+^.

**Figure:**
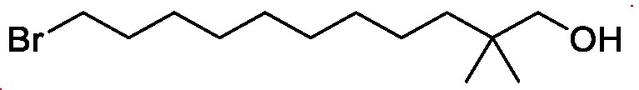

11-bromo-2,2-dimethylundecan-1-ol

Methyl 11-bromo-2,2-dimethylundecanoate (0.37 g, 1.2 mmol) was dissolved in THF (10 mL), LiAlH_4_ (0.07 g, 1.8 mmol) was added to the mixture and the reaction was stirred for 30 min at room temperature. EtOAc (5 mL) was added followed by water (2 mL), the reaction mixture was stirred for 30 min at room temperature, filtered through Celite and washed with EtOAc. The filtrate was concentrated *in vacuo* to afford the title compound (0.30 g, 88%) as an oil without further purification.

^1^HNMR (CDCl_3_) δ 3.42–3.39 (t, *J* = 6.77 Hz, 2H), 3.31 (s, 3H), 1.89–1.82 (q, *J* = 6.97 Hz, 2H), 1.44–1.38 (t, *J* = 7.1 Hz, 2H), 1.28–1.19 (m, 14H), 0.86 (s, 6H); MS *m/z* 274.40 (MH)^+^.

**Figure:**
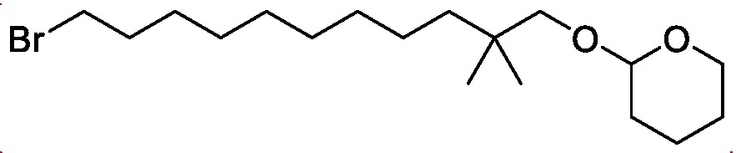

2-((11-bromo-2,2-dimethylundecyl)oxy)tetrahydro-2*H*-pyran

3,4-Dihydropyran (1.95 g, 23.0 mmol) was added to a stirred solution of 11-bromo-2,2-dimethylundecan-1-ol (4.3 g, 15.4 mmol) and camphor sulfonic acid (0.23 g, 0.92 mmol) in Et_2_O (30 mL). The reaction was stirred at room temperature for 24 h and then quenched with saturated K_2_CO_3_ (aq.) and extracted with Et_2_O (3 × 10 mL). The organic layers were dried over MgSO_4_ and concentrated. Purification with flash chromatography eluting with DCM yielded the title compound (4.32 g, 77%) as a yellow oil.

^1^HNMR (CDCl_3_) δ 4.56–4.53 (t, *J* = 3.28 Hz, 2H), 3.87–3.81 (td. *J* = 8.44 Hz, 2.96 Hz, 1H), 3.52–3.50 (m, 1H), 3.46–3.44 (d, *J* = 9.08 Hz, 1H), 3.42–3.39 (t, *J* = 6.89 Hz, 2H), 2.99–2.96 (d, *J* = 9.12 Hz, 1H), 1.89–1.84 (m, 2H), 1.72–1.66 (m, 1H), 1.64–1.49 (m, 6H), 1.48–1.39 (m, 2H), 1.32–1.22 (m, 11H), 0.89–0.87 (d, *J* = 2.61 Hz, 6H); MS *m/z* 274.30 (MH-THP)^+^.

**Figure:**
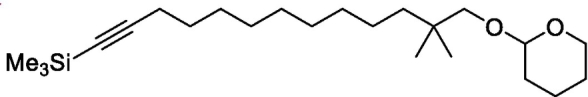

(12,12-dimethyl-13-((tetrahydro-2*H*-pyran-2-yl)oxy)tridec-1-yn-1-yl)trimethylsilane

*n*-BuLi (6.4 mL, 2 M in cyclohexane, 12.7 mmol) was added dropwise to a solution of trimethylsilyl acetylene (1.25 g, 12.7 mmol) in THF (35 mL) at −60°C. The reaction was then warmed to 0°C for 30 min. The reaction was then cooled to −20°C and HMPA (9 mL) was added dropwise, followed by 2-((11-bromo-2,2-dimethylundecyl)oxy)tetrahydro-2*H*-pyran (4.32 g, 11.9 mmol). The reaction was stirred at 0°C for 5 h, then at room temperature for 12 h. The reaction was quenched with saturated NH_4_Cl (aq.) and extracted with EtOAc (3 × 20 mL). The organic layers were dried over MgSO_4_ and concentrated. Purification with flash chromatography eluting with DCM yielded the title compound (4.0 g, 88%) as a yellow oil.

^1^HNMR (DMSO) *δ* 4.50–4.49 (d, *J* = 3.4 Hz, 1H), 3.74–3.68 (td, *J* = 8.48, 3.04 Hz, 1H), 3.44–3.40 (m, 1H), 3.36–3.33 (d, *J* = 9.08 Hz, 1H), 2.95–2.93 (d, *J* = 9.08 Hz, 1H), 2.20–2.15 (t, *J* = 6.84 Hz, 2H), 1.62–1.59 (m, 2H), 1.57–1.56 (m, 1H), 1.48–1.42 (m, 6H), 1.24–1.20 (m, 13H), 0.84 (s, 6H), 0.10 (s, 9H); MS *m/z* 381.30 (MH)^+^.

**Figure:**
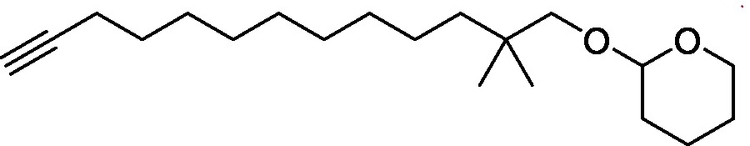

2-((2,2-dimethyltridec-12-yn-1-yl)oxy)tetrahydro-2*H*-pyran

(12,12-Dimethyl-13-((tetrahydro-2*H*-pyran-2-yl)oxy)tridec-1-yn-1-yl)trimethylsilane (4 g, 10.5 mmol) was dissolved in THF (30 mL), TBAF (15.8 mL, 1 M in THF, 15.8 mmol) were added and the reaction mixture was stirred at room temperature for 48 h. The reaction mixture was diluted with EtOAc and washed with saturated NaHCO_3_ (aq.). The organic layers were dried over MgSO_4_ and concentrated. Purification with flash chromatography eluting with DCM yielded the title compound (3.3 g, 100%) as a yellow oil.

^1^HNMR (DMSO) δ 4.50–4.49 (m, 1H), 3.74–3.67 (td, *J* = 8.49 Hz, 3.32 Hz, 1H), 3.44–3.39 (m, 1H), 3.35–3.33 (d, *J* = 9.13 Hz, 1H), 2.95–2.93 (d, *J* = 9.13 Hz, 1H), 2.15–2.11 (td, *J* = 6.88 Hz, 2.68 Hz, 1H), 1.73–1.69 (m, 1H), 1.63–1.58 (m, 1H), 1.52–1.42 (m, 5H), 1.24–1.17 (m, 15H), 0.85 (s, 6H); MS *m/z* 309.40 (MH)^+^.

**Figure:**
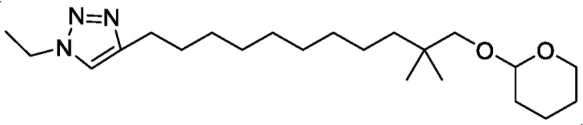

4-(10,10-dimethyl-11-((tetrahydro-2*H*-pyran-2-yl)oxy)undecyl)-1-ethyl-1*H*-1,2,3-triazole

Sodium azide (2.86 g, 44 mmol) was added to a stirred solution of ethyl iodide (3.4 g, 22 mmol) in THF/H_2_O (45/5 mL). The reaction was heated to 50°C for 1.5 h and then filtered and water (10 mL) was added to it. To the reaction mixture were added CuSO_4_.5H_2_O (0.14 g, 0.5 mmol), TBTA (0.4 g, 0.7 mmol), sodium ascorbate (0.32 g, 1.6 mmol) and 2-((2,2-dimethyltridec-12-yn-1-yl)oxy)tetrahydro-2*H*-pyran (3.39 g, 11 mmol). The reaction was then heated to 50°C for 12 h. The layers were separated, the organic layer was washed with water and the aqueous layers extracted with Et_2_O(2 × 30 mL). The organic layers were dried over MgSO_4_ and concentrated. Purification with flash chromatography eluting with DCM/EtOAc(0–45%) yielded the title compound (3.36 g, 80%) as a yellow oil.

^1^HNMR (DMSO) δ 7.84 (s,1H), 4.50–4.49 (m, 1H), 4.33–4.28 (q, *J* = 7.32 Hz, 2H), 3.73–3.67 (td, *J* = 8.48 Hz, 3.0 Hz, 1H), 3.43–3.39 (m, 1H), 3.35–3.33 (d, *J* = 9.13 Hz, 1H), 2.95–2.92 (d, *J* = 9.13 Hz, 1H), 2.60–2.56 (t, *J* = 7.52 Hz, 2H), 1.73–1.68 (m, 1H), 1.62–1.54 (m, 3H), 1.52–1.45 (m, 3H), 1.42–1.39 (t, *J* = 7.32 Hz, 3H), 1.32–1.20 (m, 15H), 0.83 (s, 6H); MS *m/z* 380.40 (MH)^+^.

**Figure:**
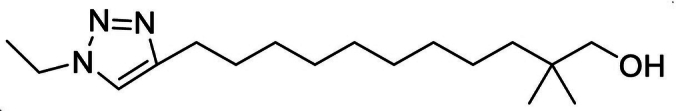

11-(1-ethyl-1*H*-1,2,3-triazol-4-yl)-2,2-dimethylundecan-1-ol

4-(10,10-Dimethyl-11-((tetrahydro-2*H*-pyran-2-yl)oxy)undecyl)-1-ethyl-1*H*-1,2,3-triazole (1.27 g, 3.4 mmol) was dissolved in DCM (30 mL), TFA (5 mL) was added to the reaction mixture which was subsequently stirred at room temperature for 48 h. The reaction was then cooled to 0°C, saturated K_2_CO_3_ (aq.) was added and the aqueous layer was extracted with EtOAc (3 × 20 mL). The combined organic extracts were dried over MgSO_4_ and concentrated. Purification with flash chromatography eluting with DCM/EtOAc(0–50%) yielded the title compound (3.36 g, 74%) as a yellow oil.

^1^HNMR (DMSO) δ 7.84 (s, 1H), 4.33–4.28 (q, *J* = 7.32 Hz, 3H), 4.11 (s, 2H), 2.60–2.56 (t, *J* = 7.56 Hz, 2H), 1.41–1.38 (t, *J* = 7.32 Hz, 3H), 1.27–1.19 (m, 16H), 0.89 (s, 6H), MS *m/z* 296.30 (MH)^+^.

**Figure:**
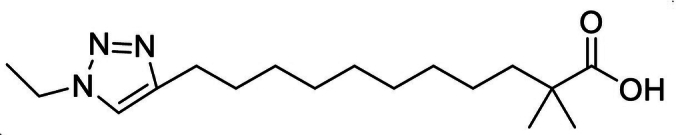

11-(1-ethyl-1H-1,2,3-triazol-4-yl)-2,2-dimethylundecanoic acid

A mixture of TEMPO (0.03 g, 1.7 mmol), NaClO_2_ (0.89 g, 9.9 mmol), 11-(1-ethyl-1*H*-1,2,3-triazol-4-yl)-2,2-dimethylundecan-1-ol (0.73 g, 2.5 mmol), NaOCl (aq.) (0.24 mL, 0.2 M, 0.05 mmol), were dissolved in MeCN (20 mL). Buffer was formed by dissolving NaH_2_PO_4_.H_2_O (0.26 g, 1.6 mmol) and Na_2_HPO_4_ (0.23 g, 1.6 mmol) in H_2_O (24 mL) and added to the reaction mixture. The reaction mixture was stirred at 50°C over 48 h. The reaction was diluted with brine and extracted with EtOAc (3 × 20 mL). The combined organic extracts were dried over MgSO_4_ and concentrated to yield the title compound in quantitative yield without further purification.

^1^HNMR (DMSO) δ 12.01 (br.s, 1H). 7.83 (s, 1H), 4.34–4.28 (q, *J* = 7.32 Hz, 2H), 2.58–2.56 (t, *J* = 7.53 Hz, 2H), 1.62–1.52 (m, 3H), 1.41–1.37 (t, *J* = 7.32 Hz, 3H), 1.36–1.12 (m, 13H), 0.76 (s, 6H); ^13^CNMR (DMSO) δ 178.80, 146.86, 121.06, 69.82, 44.23, 41.19, 34.70, 30.13, 29.52, 29.08, 28.98, 28.88, 28.77, 28.72, 28.57, 15.38; MS *m/z* 310.30 (MH)^+^, Calculated for C_17_H_31_N_3_O_2_ (MH^+^) 310.24890, found 310.24958.

### 2.3 Osteoclast cultures from bone marrow cells

As previously described (Cornish et al., 2001), bone marrow cells were collected, by flushing with αMEM, from the cavity of the femurs and tibias of CD-1 male mice aged 4–6 weeks. After pre-incubation for 2 h, non-adherent cells were collected and seeded in the 48-well plates at 5 × 10^5^ cells/0.5 mL/well in 10% FBS/αMEM (Day 0). Osteoclastogenesis was stimulated by 1,25-dihydroxyvitamin D_3_ [1,25(OH)_2_D_3_] added on Days 0, 2 and 4 at the final concentration of 10^−8^ M. Test factors and vehicle were added on Days 2 and 4. When the test factors were dissolved in methanol, the final concentration of methanol was 0.5% in the culture medium. Control groups were also treated with 0.5% methanol in the culture medium. At the end of culture (Day 7), the cells were fixed and stained for tartrate-resistant acid phosphatase (TRAP) using a kit from Sigma (387-A). TRAP positive cells with 3 or more nuclei were defined as osteoclasts and scored under light microscopy.

### 2.4 Complexation of tetrazole analogue and β-CD

This was referred to the previous report (Ono-Moore et al., 2018). The analogue and β-CD (Sigma) were initially dissolved in methanol at a molar ratio of 1:1.5 unless otherwise indicated. The mixture was dried at 40°C under sterile conditions and then reconstituted in H_2_O with shaking for a few hours under room temperature. This complex of analogue and β-CD was used in the calvarial local injection model *in vivo*.

### 2.5 Bone marrow cell viability assay

The cell culture conditions are the same as those for osteoclastogenesis assay described above in Section 2.3. Twenty-four hours after the treatment on day 2 of culture, alamarBlue™ (Thermo Fisher, NZ) was added to the cells (50 μL/well) and continued to incubate for 4 h. The medium was then measured for fluorescence at 590 nm by using Synergy 2 Multi-Detection Microplate Reader (BioTek, USA).

### 2.6 Real-time PCR

Total RNA was extracted from the bone marrow cells cultured in the same conditions as those in the osteoclastogenesis assay above (in Section 2.3) by using the RNeasy Mini Kit (Qiagen) with the treatment of RNase-free DNase (Qiagen) according to the manufacturer's instruction. First strand cDNA was synthesized by reverse transcription using random priming and SuperScript III reverse transcriptase (Thermo Fisher, NZ). Multiplex real-time PCR was conducted in a 384-well optical reaction plate by using TaqMan master mix kit (Applied Biosystems) and ABI PRISM 7900HT sequence detection system (Applied Biosystems). VIC-labelled probes were used for endogenous control 18S rRNA and FAM-labelled probes were used for the genes of interest. ∆∆Ct method was used to calculate the relative gene expression levels. All the reagents were purchased from Thermo Fisher.

### 2.7 Viability assays with primary osteoblasts and MC3T3-E1 cells

Rat primary osteoblasts were prepared as previously described (Cornish et al., 1999). Briefly, calvariae were dissected out aseptically from 20-day fetal Wistar rats (mixed sexes). After suture and periosteal tissue being removed, the frontal and parietal bones were collected and subjected to sequential digestion with collagenase. The cells collected from digestions 3 and 4 were washed with 10% FBS/DMEM and cultured in the same medium in T75 flasks until 90% confluency. Primary osteoblasts were seeded in 24-well plates at the density of 2.5 × 10^4^ cells/well in 5% FBS/MEM containing 5 μg/mL ascorbic acid-2-phosphate. MC3T3-E1 cells were seeded at 4 × 10^4^ cells/well in 5% FBS/αMEM. After culture for 24 h, rat primary osteoblasts or MC3T3-E1 cells were subjected to serum starvation for 24 h followed by the treatment of the test factors for another 24 h. At this time point, alamarBlue™ was added and measured as described above.

### 2.8 *In vivo* studies

**Fig. 2:**
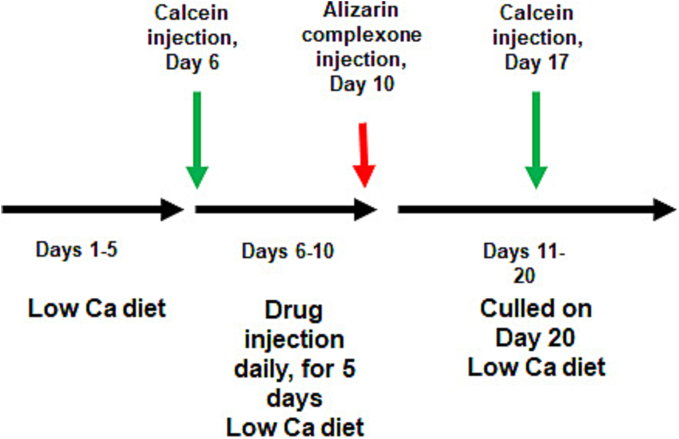
Treatment regime for the tetrazole analogue locally injected in the hemi-calvaria in mice. The mice were fed low calcium diet from Days 1 to 20. The analogue was injected once a day from Day 6 for five consecutive days. The calvaria bone was labelled with the green dye calcein by injection twice on Days 6 and 17 and labelled with the red dye Alizarin complexone on Day 10 by injection.

As described previously (Cornish et al., 1993, Cornish et al., 1997a), male CD-1 mice aged 9-weeks old were randomly grouped (15 mice/group) and switched to the low calcium diet (0.14% calcium, Cat # SF05-065, Specialty Feeds, Australia) 5 days before the injection and throughout the experimental period, to boost osteoclast activity. The tetrazole analogue complexed with β-CD or vehicle (equivalent β-CD) was injected over the periosteum of the right hemicalvaria daily for 5 consecutive days at the dosage indicated (50 μL/injection). Calcein (30 mg/kg) was injected twice: on the first day of the tetrazole analogue injection and 7 days after the last analogue injection. Calcein was used to label the mineral deposition at the two time points and the interval between the two green labels was used in the calculation of the bone formation parameters. Alizarin complexone (20 mg/kg) was injected on the last day of the analogue injection to separate the two calcein labels (Fig. 2). The animals were weighed before the first injection and before culling (10 days after the last injection) as an indication of the health of the animals. All mice were maintained in our specific pathogen-free facilities. The calvariae were dissected out, fixed in 4% paraformaldehyde/PBS and stored at 4°C (in sucrose). The calvarial bones were embedded in the optical cutting temperature (OCT) compound and sectioned at 7 μm by using CryoStar NX70 (Thermo Fisher). The sections were examined for the bone formation parameters under fluorescent microscope (Olympus BX50) with the use of the program OsteoMeasure™ (OsteoMetrics, Atlanta, GA). To examine for the osteoclastic parameters, the sections were decalcified in 10% DETA for 24 h at 4°C and then stained for TRAP with the same kit used for the cultured osteoclast staining. The TRAP-stained sections were scored under the microscope with the use of the program CellSens (Olympus). The assessor was blindfold to the sample identities.

### 2.9 Statistical analyses

Data are presented as mean ± SEM from a representative experiment. Statistical analysis was performed using Prism version 8 (GraphPad Software, Inc.) and the significance between the groups was determined by either *t-*test or ANOVA with Dunnett's *post-hoc* test. *P* < 0.05 was considered as significantly different.

## 3 Results

### 3.1 *In vitro* osteoclastogenesis assay

**Fig. 3:**
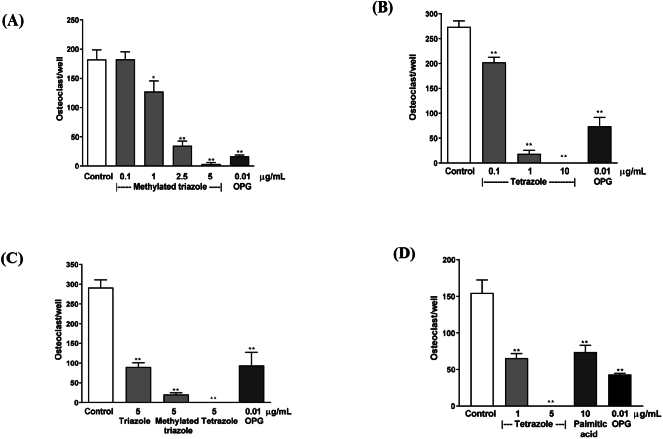
Inhibition of osteoclastogenesis by the analogues and palmitic acid, and potency comparison in mouse bone marrow cultures. The molecular weight (MW) of the compounds and their equivalent molar concentrations at 5 μg/mL are as follows: triazole MW: 281.4 (17.8 μM); methylated triazole MW: 309 (16.2 μM); tetrazole MW: 319 (15.7 μM); palmitic acid MW: 256.4 (19.5 μM). OPG is as a positive control. Data are presented as Mean ± SEM; *: *P* < 0.05; **: *P* < 0.01, Dunnett's test.

The analogues synthesized were based on natural palmitic acid (Fig. 1A) as the backbone. Based on the molecular structure of the previous analogues showing improved potency, particularly triazole (Marshall et al., 2013), two more molecules were produced. The first one is a methylated triazole (Fig. 1B) with two methyl groups added at the α position of the triazole carbon chain. The second one is a tetrazole compound, in which the carboxylic acid group is replaced by a tetrazole bioisostere (Fig. 1C). In the osteoclastogenesis assay with mouse bone marrow cell cultures, both methylated triazole and tetrazole analogues displayed higher inhibitory activity than the earlier synthesized triazole, with significant effects seen at 1 or 0.1 μg/mL, respectively (Fig. 3A and B). At concentrations of 5 μg/mL or greater, osteoclastogenesis was completely blocked by the tetrazole analogue, which has displayed much higher activity than any triazole analogue and is superior to natural palmitic acid with 10-fold higher potency (Fig. 3C and D). Importantly, while scoring the osteoclast number, attention has also been paid to the stromal cells and no obvious change in stromal cell population was seen within the concentrations tested by visual inspection in this bone marrow culture.

**Fig. 4:**
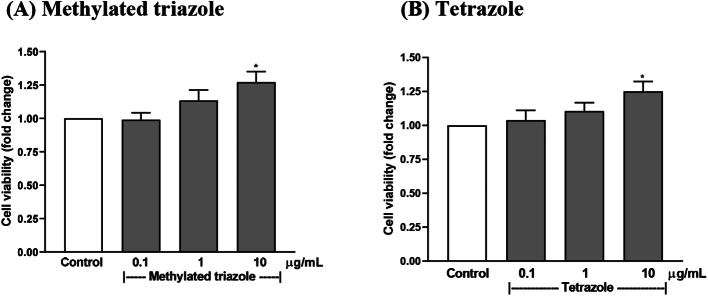
Viability assays of the analogues in mouse bone marrow cells. Methylated triazole (A) and tetrazole (B) analogues in the assays with mouse bone marrow cells. Data are presented as Mean ± SEM; *: *P* < 0.05, Dunnett's test.

To verify that the inhibitory effect on osteoclastogenesis is not due to non-specific cytotoxicity, the analogues were tested for their effect on the viability of bone marrow cells. The results show that the analogues did not reduce the cell viability but rather stimulated it. In these assays, modest stimulation was seen for both the analogues at 10 μg/mL. No significant change was seen below this concentration (Fig. 4A and B). These results are consistent with the direct inspection in the osteoclastogenesis assay with bone marrow cultures, in which no obvious change was seen in stromal cell population. This has demonstrated that the analogues are not cytotoxic within the concentrations tested. Since the tetrazole analogue was the most potent analogue, further study has concentrated on this compound.

### 3.2 Gene expression

**Fig. 5:**
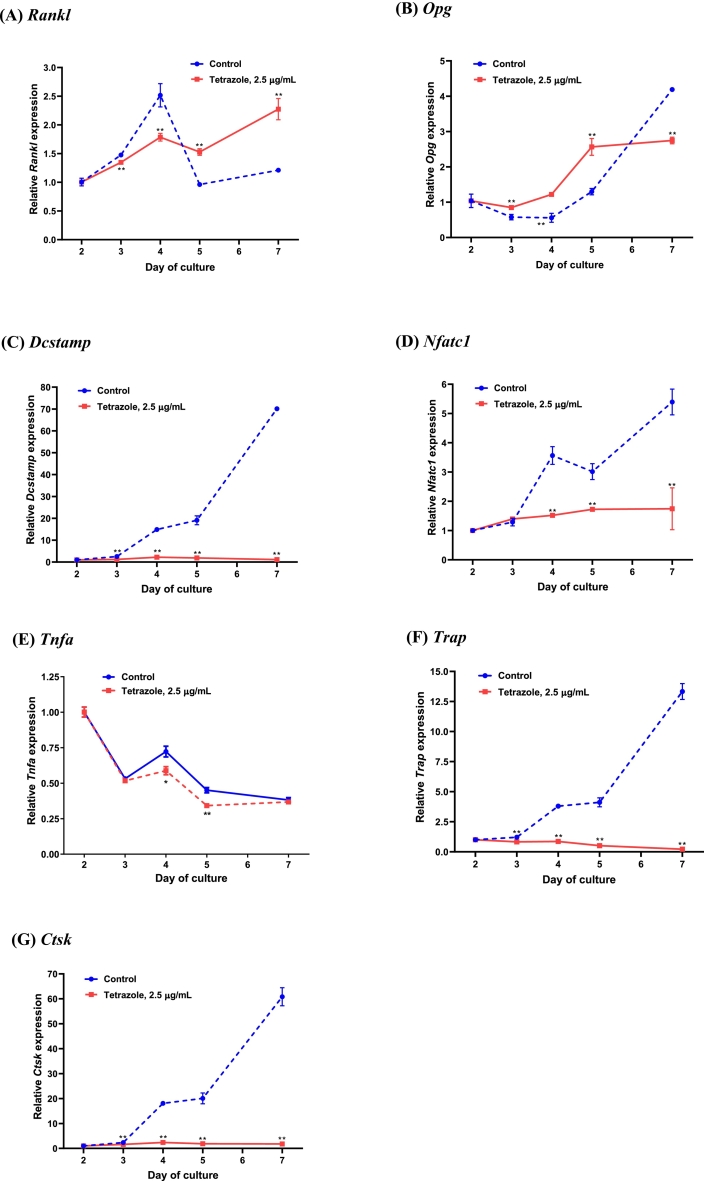
Osteoclastic gene expression in mouse bone marrow cells treated with the tetrazole analogue. The bone marrow cells were treated with the tetrazole analogue on days 2 and 4, and RNA collected on the time points indicated. Gene expression was measured by real-time PCR. Data are presented as Mean ± SEM; *: *P* < 0.05; **: *P* < 0.01, *t*-test.

To probe the mechanisms of action of tetrazole analogue on osteoclasts, its effect on the expression of the key osteoclastogenic/osteoclastic marker genes have been investigated. While RANKL and OPG play predominant roles in regulating osteoclast maturation, their expression levels were measured upon the treatment of the analogue. Fig. 5 shows that the changes in *Rankl* and *Opg* expression were not consistent with the reduction in osteoclastogenesis, suggesting that the effect of the analogue is independent of these two molecules. Then the expression profiles of *Dcstamp* and *Nfatc1* and *Tnfa* were studied. DC-STAMP is a major molecule involved in cell-cell fusion, a key process during osteoclast maturation (Iwasaki et al., 2008), while NFATc1 plays a role as a pivotal transcription factor regulating the genes responsible for osteoclast development (Zhao et al., 2010). TNF-α alone is known to be able to induce osteoclast formation independent of RANKL signalling and the induction is not inhibited by OPG (Fuller et al., 2002). Consistent with suppression in osteoclast number, the analogue greatly reduced the expression of *Dcstamp* and *Nfatc1* while mildly reduced the level of *Tnfa*. In the meantime, the expression of *Trap* and *Ctsk*, markers for osteoclasts, was also dramatically reduced (Fig. 5).

**Fig. 6:**
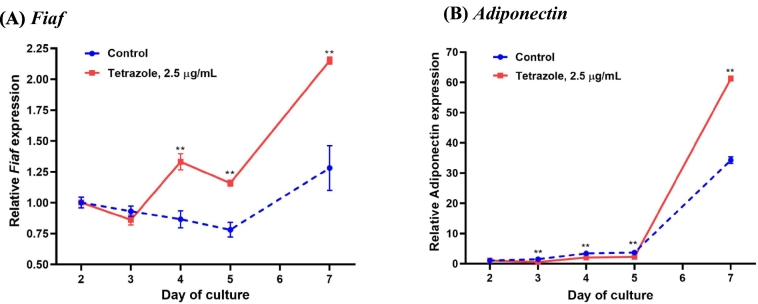
Stimulation of FIAF and adiponectin gene expression by the tetrazole analogue. The bone marrow cells were treated with the tetrazole analogue on days 2 and 4, and RNA collected on the time points indicated. Gene expression was measured by real-time PCR. Data are presented as Mean ± SEM; **: *P* < 0.01, *t*-test.

To see if the tetrazole analogue retains the features of natural fatty acids, it was tested for its effect on the expression of the genes encoding fasting-induced adipose factor (FIAF) and adiponectin, since both of the adipokine genes are the distinctive targets for natural fatty acids (Miida and Hirayama, 2010; Banga et al., 2009). As expected, the expression of both genes was characteristically elevated by the analogue (Fig. 6).

### 3.3 On osteoblast viability

**Fig. 7:**
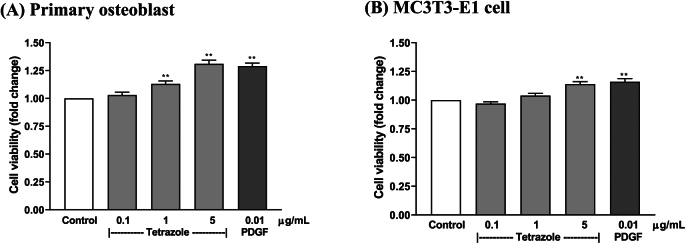
The tetrazole analogue enhanced cell viability in rat primary osteoblasts (A) and osteoblastic MC3T3-E1 cells (B). Platelet-derived growth factor (PDGF) as a positive control. **: *P* < 0.01, Dunnett's test.

The above results have shown that the tetrazole analogue elevated the viability in bone marrow cells, which contain majority of osteoblastic stromal cells. To confirm the effect on osteoblasts, the analogue was further tested in rat primary osteoblasts isolated from calvaria and in osteoblastic MC3T3-E1 cell line. The results show that the analogue also stimulated the cell viability in both of the osteoblast culture assays (Fig. 7).

### 3.4 *In vivo* study

**Fig. 8:**
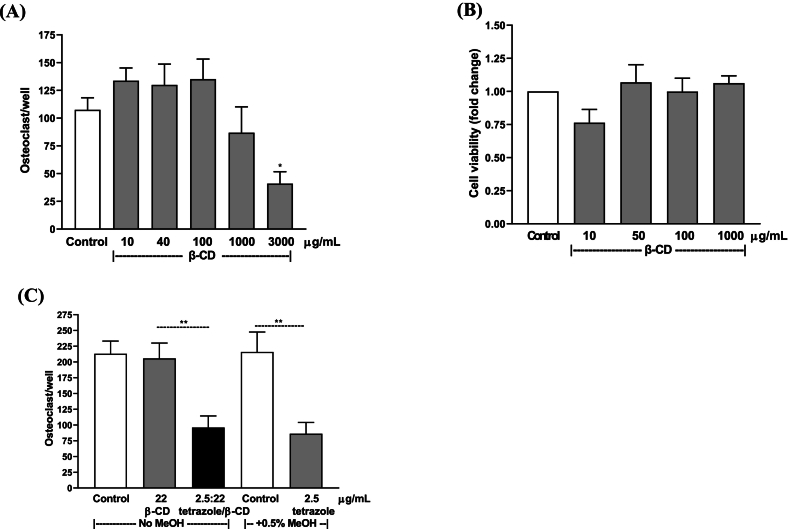
Effect of β-CD, the tetrazole analogue and the tetrazole/β-CD complex in cultured mouse bone marrow cells. (A) and (B): β-CD on osteoclastogenesis and viability, respectively. (C) Potency comparison of osteoclast inhibition by the tetrazole analogue alone and the tetrazole/β-CD complex. The tetrazole/β-CD complex (1:2 molar ratio) was dissolved in water, while the un-complexed tetrazole analogue was dissolved in methanol with the final concentration of methanol at 0.5% in the culture medium. Data are presented as Mean ± SEM; *: *P* < 0.05, Dunnett's test. **: *P* < 0.01, *t*-test.

The anabolic effect of the tetrazole analogue *in vitro* prompted us to test its effect on bone homeostasis *in vivo*. In this assay, the most potent analogue-tetrazole, was tested in a calvarial local injection model. Since the analogue *per se* is still not soluble in water, in order to avoid using organic solvents which are toxic to animals if injected, the analogue was complexed to the carrier β-CD, which is safe in animals and humans. In addition, it has no significant effect on osteoclast formation and bone marrow cell viability at concentrations up to 1000 μg/mL as tested *in vitro* (Fig. 8A and B). The activity of the complexed tetrazole/β-CD (at 1:2 molar ratio) in inhibiting osteoclastogenesis was validated in bone marrow culture, being comparable to the activity of the un-complexed tetrazole analogue dissolved in methanol (Fig. 8C).

**Fig. 9:**
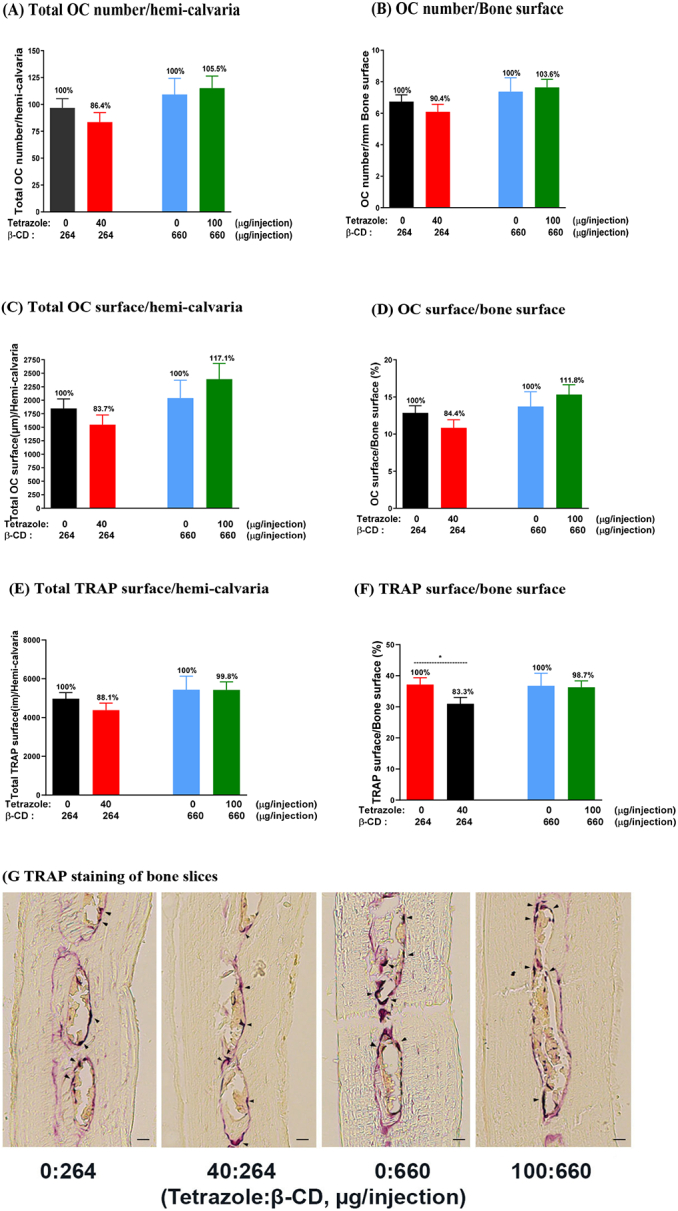
Osteoclastic parameters in mouse calvaria injected with β-CD alone and the tetrazole/β-CD complex. Mice were injected with two doses of β-CD (264 and 660 μg/injection) in the control groups and two doses of the tetrazole/β-CD complex (40/264 and 100/660 μg/injection) in the treatment groups. Sections of the hemicalvaria of the injected side were scored (4 sections/each hemi-calvaria) after TRAP staining. Osteoclasts were indicated by the black arrows. Scale bar = 20 μm. Data are presented as Mean ± SEM; *: *P* < 0.05, *t*-test.

**Fig. 10:**
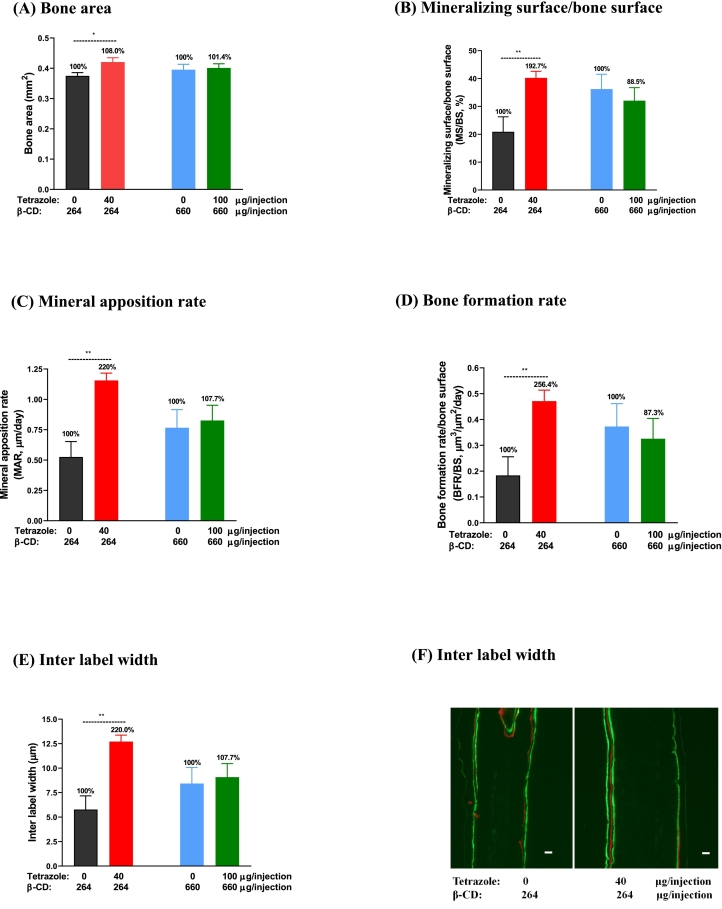
The tetrazole analogue increases bone formation in mouse calvaria. Mice were injected with two doses of β-CD (264 and 660 μg/injection) in the control groups and two doses of the tetrazole/β-CD complex (40/264 and 100/660 μg/injection) in the treatment groups. (F) Green: label with calcein; Red: label with alizarin complexone. Inter-label width was measured between the two green labels. Scale bar = 20 μm. Data are presented as Mean ± SEM; *: *P* < 0.05, **: *P* < 0.01, *t*-test. The difference between two control dosages (264 and 660 μg β-CD/injection) is not statistically significant (*P* > 0.05, *t*-test) in each of the parameters.

Fig. 2 illustrates the *in vivo* study plan, in which the animals were fed low calcium diet to stimulate osteoclast activity and subcutaneously injected with the test factor or vehicle for 5 consecutive days. In this assay, each of the two vehicle control groups were administered 264 and 660 μg β-CD/injection, respectively, which are the equivalent dosage of the carrier in the two treatment groups (40:264 and 100:660 μg/injection, tetrazole:β-CD). The results show that the analogue at lower dosage significantly reduced the osteoclastic parameter TRAP surface/bone surface. TRAP surface was measured as the TRAP released from osteoclasts and deposited on the bone surface plus the osteoclast surface which was also stained TRAP positive. The other osteoclastic parameters in total osteoclast number/hemicalvaria, osteoclast number/bone surface, total osteoclast surface/hemicalvaria, osteoclast surface/bone surface and total TRAP surface/hemicalvaria were also measured, but significant difference was not obtained (Fig. 9). Meanwhile, bone formation parameters were increased at the same dosage. Significant increases were seen in bone area, mineralizing surface/bone surface, mineral apposition rate and bone formation rate as compared to the vehicle group (264 μg/injection β-CD). The administration of the analogue also greatly increased the width between the two calcein labels in the bone (Fig. 10). All these parameters indicate that the analogue has stimulated calvarial bone growth. However, these anabolic effects were not seen at the higher dosage of the complex with 100 μg tetrazole analogue and 660 μg β-CD per injection.

**Fig. 11:**
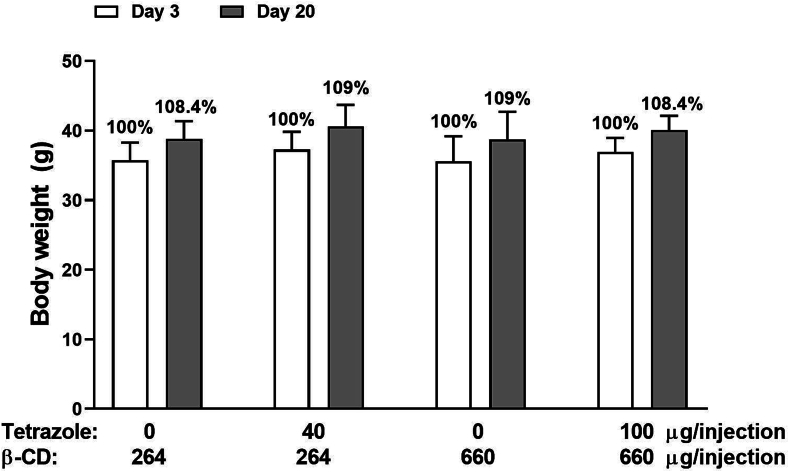
Mouse body weight was not changed by either β-CD alone or the tetrazole/β-CD complex. Mice were injected with two doses of β-CD (264 and 660 μg/injection) in the control groups and two doses of the tetrazole/β-CD complex (40/264 and 100/660 μg/injection) in the treatment groups. Body weight was measured on Days 3 and 20.

To monitor the health of the animals, their body weight was measured on days 3 and 20. Neither the vehicle nor the analogue/β-CD complex changed the weight of the animals (Fig. 11).

## 4 Discussion

The present results demonstrate that the analogues have substantially higher osteoclast inhibition potency than their natural counterpart. While the signalling pathways for fatty acids are still not clear, it is known that fatty acids can function intracellularly by being taken up by cells and act extracellularly by interacting with cell surface receptors. This suggests that the elevated activity of the analogues could result from either improved intracellular or extracellular signalling, or both.

The main strategy of the analogue development is to increase its stability inside the cells according to the intracellular fates of fatty acids. Free fatty acids could traverse the cell membrane either *via* passive diffusion or *via* the mediation of protein carriers (Doege and Stahl, 2006). Once taken up by the cells, they are firstly activated to form acyl-CoA through the step catalysed by acyl-CoA synthetase (ACS) (Balaban et al., 2015; Kushwaha et al., 2018). Then they are subject to either of two fates: esterification (60–70%) in endoplasmic reticulum to form glycerides and phospholipids, or β-oxidation (20–30%) in mitochondria (Balaban et al., 2015; Carta et al., 2017). During β-oxidation, a fatty acid is broken down between the α and β positions of the chain giving rise to a two-carbon metabolite acetyl-CoA, which enters the TCA cycle to generate ATP and ends up with CO_2_ (Kushwaha et al., 2018).

Many studies show that labelled free palmitic acid is rapidly cleared *via* esterification and β-oxidation. In rat retinas, 80% of ^3^H-palmitic acid was incorporated into phospholipids 2 h after injection (Wetzel and O'Brien, 1986). In pigs infused with ^14^C-palmitic acid, the cumulative ^14^CO_2_ recovered from expiration for 6 hours post-treatment comprised 19% of the total radioactivity administered (Boyd et al., 1982). Considering that the recovery rate of ^14^CO_2_ could not be 100%, the ^14^C-palmitic acid subjected to β-oxidation should be higher than 19%. Rapid metabolism of palmitic acid has also been documented in the studies *in vitro*. In isolated rat cardiac myocytes, nearly 80% of palmitic acid was esterified or oxidized within 3 min (Luiken et al., 1997). In cultured rat hepatocytes, the turnover rate of the fatty acid is slower, but nearly 40% was metabolized after 4 h (Rioux et al., 2000). Though the metabolism rate of the fatty acid in bone cells is yet to be determined, it could be reasonably inferred that rapid metabolism limits the activity of fatty acids on osteoclast inhibition based on the above studies. Therefore, it was our aim to reduce the esterification and oxidation rates by modifying the molecule to produce more stable compounds with higher potency.

The results from the current study indicate that the modification of palmitic acid has successfully elevated the activity in inhibiting osteoclastogenesis *in vitro* and positively regulating bone homeostasis *in vivo*. Based on our previous (Marshall et al., 2013) and current assays *in vitro*, the structure-activity relationship could be summarized as follows: (1) The analogue with the triazole group farthest away from the acid group displays the highest potency; (2) Insertion of two methyl groups in the α position in the chain improves the activity on top of triazole insertion in the farthest position; (3) Replacement of the acid group with a tetrazole group greatly elevates the activity compared to the molecules with the insertion of a triazole group alone and with the insertion of both the triazole and methyl groups. Insertion of a triazole group is a common chemical strategy to produce a biologically active compound. The introduction of this unit provides the molecule with an aromatic character and rigidity, and better solubility with the reduction in *clog P* (Marshall et al., 2013), though it is unknown how the insertion position of the triazole group affects the biological activity. Regarding the activity of the methylated triazole, the addition of the two methyl groups in the α position could likely block β-oxidation, in which the carbon-chain is degraded between α and β positions giving rise to a two-carbon molecule, acetyl CoA (Houten et al., 2016). While fatty acid esterification requires the involvement of the carboxylic acid group, the replacement of the carboxylic acid group with the tetrazole group could obviously block this process and stabilize the molecule. In a future search for even more potent molecules, it would be worth trying the combination of the above three groups (triazyl, methyl and tetrazyl) in a single molecule.

In addition to being more resistant to catabolism inside the cells, the possibility could not be excluded that the analogues could also function more efficiently before entering the cells *via* interaction with the cell surface receptors due to the change in their chemical structure and conformation. The cell surface receptors for fatty acids include G-protein-coupled receptors 40 (GPR40), 41 (GPR41), 43 (GPR43) and 120 (GPR120), the last of which is reported to mediate long chain fatty acid signalling (Morgan and Dhayal, 2009; Ahn et al., 2016). Our earlier study also shows that GPR120 is predominantly expressed in osteoclastic RAW264.7 cells (Cornish et al., 2008), though its actual involvement in fatty acid signalling still needs to be further clarified. From this point of view, it is possible that the analogues, like palmitic acid, interacts with the cell surface receptor, but with higher efficiency leading to higher activity.

Previous reports from other groups show that palmitic acid stimulated rather than inhibited osteoclastogenesis (Drosatos-Tampakaki et al., 2014; Tao et al., 2024), contrary to our previous and current findings. This discrepancy could be due to the difference in culture conditions such as BSA addition, osteoclastogenic stimulators used, cell density seeded and particularly palmitic acid concentrations. The concentrations of palmitic acid used in our studies were 5–10 times lower than those used by the other group in the studies *in vitro*. In the *in vivo* studies, we found that the tetrazole analogue displayed anabolic effect on bone, while the other group found that palmitic acid behaved in the opposite way (Tao et al., 2024). It is noted that the dosage of palmitic acid used (100 mg/kg/day in rat) was over 200 times higher than the dosage of the analogue (40 μg/injection/mouse/day in mouse) used in our study. In addition, the tetrazole analogue may not completely mimic the biological properties as palmitic acid due to the structural modification. These concentration and structural factors may have accounted for the different effects observed in the different studies on osteoclasts. It has also been documented that palmitic acid induced cell death in osteoblasts (Gunaratnam et al., 2013, Gunaratnam et al., 2014), while the analogues in the current study stimulated bone marrow cells and osteoblast viability at much lower concentrations. This difference in the observed effects could also be due to the concentrations used and the structural modification of the molecules. The palmitic acid analogue is no longer a palmitic acid in chemical structure and their biological effects could be different.

It has been well documented that free long-chain fatty acids including palmitic acid are potent stimulator to FIAF expression *via* the activation of peroxisome proliferator-activated receptor (PPAR)-δ (Staiger et al., 2009; Gonzalez-Muniesa et al., 2011). Palmitate greatly enhanced adiponectin expression along with the promotion of mouse adipocyte differentiation (Petrighi Polidori et al., 2012). The results in Fig. 6 shows that the tetrazole analogue also promotes the expression of both adipokines. It could be inferred that the analogue still retains the biological properties of the natural fatty acid though the chemical structure has been modified. The stimulation on the expression of the adipokines also further indicates that the inhibition of the analogue on the osteoclastic genes is a specific action rather than a non-specific cytotoxic effect.

In the current *in vivo* assay, two control groups were locally injected with two doses of β-CD (264 and 660 μg/injection) while two treatment groups received two doses of complexed tetrazole/β-CD (40 and 100 μg tetrazole complexed with 264 and 660 μg β-CD, respectively, per injection). The lower dose of analogue (40 μg analogue/264 μg β-CD) displayed an anabolic effect as seen in the reduction of the osteoclastic parameters TRAP surface/bone surface and in the significant increase of all the bone formation parameters. The effect of the analogue on bone formation is remarkable with some parameters increased by about two-fold. Unexpectedly, the effect on bone formation is more pronounced than that on bone resorption, somewhat inconsistent with the observations *in vitro*, in which the analogue potently inhibits osteoclastogenesis and mildly elevated osteoblast viability. While the elevation in osteoblast viability might have partly contributed to the increased bone formation parameters, it is uncertain if the increase in bone formation also resulted from the action of the analogue on osteoclasts. It is noted that the analogue has significantly reduced the osteoclastic parameter TRAP surface/bone surface, but the other osteoclastic parameters were not significantly changed. Therefore, it remains a question to be answered if the analogue could also target osteoclasts *in vivo* as it did *in vitro*. Regarding the effect on osteoblasts, it is likely that the stimulatory effect of the analogue on the cell viability, as seen in primary osteoblasts, MC3T3-E1 cells and mouse bone marrow cells (mostly osteoblastic stromal cells), might have contributed to the increased bone formation *in vivo*. However, since this assay is not a direct determination of cell population and the colorimetric reading could be interfered by other factors (Rampersad, 2012), confirmation with more rigorous measurements will help the establishment of the osteoblastic effect on bone formation. As this project has been mainly focused on the effect of osteoclasts using an osteoclast inhibition model (bone marrow culture) to screen the analogues synthesized, the work on osteoblast has been preliminary with the viability assay employed mainly to monitor cytotoxicity. Upon the effect of the analogue on osteoblast viability *in vitro* and on bone formation *in vivo*, it is worth further looking into the osteoblastic effect of the analogue *in vitro*, such as its effects on proliferation, mineralization and on the expression of the differentiation marker genes (collagen, alkaline phosphatase and runx2, etc.).

The mice used in the *in vivo* study were 9-weeks old, close to the mature age (usually from 10 weeks old). These mice maintained some growths as seen in the increase of the body weight and thus allows for dynamic dye labelling for bone formation assay. Meanwhile, the mice at this age were also suitable for bone resorption study. These features have been confirmed in our previous studies (Cornish et al., 2004, Cornish et al., 1997b, Cornish et al., 2000) and were useful for the evaluation of the analogue, which has shown the effects on both osteoblasts and osteoclasts *in vitro*. The dynamic labelling is an indication of bone formation, which also occurs in older animals. It is likely that the results obtained from growing animals would be consistent to the effect in older animals. This was seen in the OPG study, in which the anabolic effect of OPG was seen in both the rapidly growing mice and in the adult rats (Simonet et al., 1997). It is now well known that OPG-related agent is a clinical therapy for osteoporosis in ageing subjects. Certainly, the physiological statuses in growing animal are different from adult or aged ones, in which bone metabolism is prone to negative homeostasis, inconsistency in bone effect by a regulator in different age groups could not be excluded. The results from the current study have shown the anabolic effect in the growing mice and further study with older animal model will help the evaluation of the analogue for the potential osteoporosis therapy.

It is of note that the higher dose injection (100 μg analogue/660 μg β-CD) has no significant effect on bone parameters *in vivo*. The inconsistency in dose response could be explicable. The dosage 100 μg tetrazole analogue/injection used *in vivo* were determined empirically based on the *in vitro* dosage of 2.5 μg/mL, at which no cytotoxicity was seen. This equivalent dosage translation between *in vitro* and *in vivo* has worked well in our previous studies using peptide factors (Cornish et al., 1997a, Cornish et al., 2004), which are readily cleared *in vivo*. However, the analogue tested in the current study was designed to counteract the metabolism that could otherwise degrade fatty acids. Therefore, the analogue could be accumulated around the injection site after repeated administration (daily injection for five consecutive days). The accumulation of the compound could lead to toxicity due to overdosage, which offsets the anabolic action of the analogue, particularly for the prolonged length of the treatment. This possibility is supported by the previously reported cytotoxicity in cultured osteoblasts treated with high concentrations of natural fatty acid (Gunaratnam et al., 2014).

Cyclodextrin (including β-CD) is a group of non-toxic solubilizers and has been approved by FDA to use in various areas, such as food and medicine additives (Gidwani and Vyas, 2015). It has also been used as solubilizing agent for some chemicals in research (Zhong et al., 2020). However, its use in solubilizing fatty acids in a bone study has not been documented as far as we know. In the current study, we have successfully solubilized the analogue by complexation with β-CD. It is possible that this technique could also be applicable to natural fatty acids to improve the ease of bone studies with fatty acids. Generally, aqueously insoluble fatty acids used in research are either dissolved in organic solvents or are conjugated to bovine serum albumin for better solubility (Spector et al., 1969; Gremlich et al., 1997; Thompson et al., 2000). However, this could be problematic. Organic solvents are toxic to cells and animals and are not suitable for *in vivo* study. If conjugation with bovine serum albumin is used, data interpretation is also complicated since bovine serum albumin is highly antigenic (Wahn et al., 1981). Currently, few *in vivo* studies with fatty acids have used an injection model in animals, likely due to the solubility issue of the compounds. The present study provides a technical solution to improve the study of fatty acids.

In conclusion, we have produced some analogues by modification of palmitic acid. These fatty acid analogues display much more potent activity than the natural counterpart in osteoclast inhibition *in vitro*. The highest potency was seen in an analogue in which the carboxylic acid group is replaced by a tetrazole unit and a triazole unit is inserted in the carbon chain farthest away from the tetrazole unit. This analogue, with the aid of β-CD for solubility, has also displayed anabolic effect on bone *in vivo*. This has demonstrated that modification of the natural fatty acid is a novel strategy to produce molecules with potent skeletal activity and with the potential to develop new therapies for bone disorders.

## CRediT authorship contribution statement

**Jian-ming Lin:** Methodology, Formal analysis, Investigation, Funding acquisition, Data curation, Writing – original draft, Writing – review & editing. **Ivo Dimitrov:** Methodology, Formal analysis, Investigation, Data curation, Writing – original draft, Writing – review & editing. **Karen E. Callon:** Investigation, Formal analysis, Data curation. **Maureen Watson:** Investigation, Formal analysis, Data curation. **Ian R. Reid:** Conceptualization, Writing – review & editing. **William A Denny:** Conceptualization, Project administration, Resources, Writing – review & editing, Supervision, Funding acquisition. **Jillian Cornish:** Conceptualization, Project administration, Resources, Writing – review & editing, Supervision, Validation, Funding acquisition.

## Declaration of competing interest

The authors declare that they have no known competing financial interests or personal relationships that could have appeared to influence the work reported in this paper.

## Data Availability

Data will be made available on request.
